# CD19 CAR immune pressure induces B-precursor acute lymphoblastic leukaemia lineage switch exposing inherent leukaemic plasticity

**DOI:** 10.1038/ncomms12320

**Published:** 2016-07-27

**Authors:** Elad Jacoby, Sang M. Nguyen, Thomas J. Fountaine, Kathryn Welp, Berkley Gryder, Haiying Qin, Yinmeng Yang, Christopher D. Chien, Alix E. Seif, Haiyan Lei, Young K. Song, Javed Khan, Daniel W. Lee, Crystal L. Mackall, Rebecca A. Gardner, Michael C. Jensen, Jack F. Shern, Terry J. Fry

**Affiliations:** 1 Pediatric Oncology Branch, Center for Cancer Research, National Cancer Institute, National Institutes of Health, Bethesda, Maryland 20892, USA; 2 Genetics Branch, National Cancer Institute, National Institutes of Health, Bethesda, Maryland 20892, USA; 3 Division of Oncology, The Children's Hospital of Philadelphia, and Department of Pediatrics, Perelman School of Medicine of the University of Pennsylvania, Philadelphia, Pennsylvania 19104, USA; 4 Ben Towne Center for Childhood Cancer Research, Seattle Children's Hospital, and Department of Pediatrics, University of Washington, Seattle, Washington 98105, USA

## Abstract

Adoptive immunotherapy using chimeric antigen receptor (CAR) expressing T cells targeting the CD19 B lineage receptor has demonstrated marked success in relapsed pre-B-cell acute lymphoblastic leukaemia (ALL). Persisting CAR-T cells generate sustained pressure against CD19 that may drive unique mechanisms of resistance. Pre-B ALL originates from a committed pre-B cell or an earlier progenitor, with potential to reprogram into other hematopoietic lineages. Here we report changes in lineage markers including myeloid conversion in patients following CD19 CAR therapy. Using murine ALL models we study the long-term effects of CD19 CAR-T cells and demonstrate partial or complete lineage switch as a consistent mechanism of CAR resistance depending on the underlying genetic oncogenic driver. Deletion of *Pax5* or *Ebf1* recapitulates lineage reprogramming occurring during CD19 CAR pressure. Our findings establish lineage switch as a mechanism of CAR resistance exposing inherent plasticity in genetic subtypes of pre-B-cell ALL.

## Abstract (web summary)

CAR-T targeting CD19 have been successfully used in a variety of B-cell malignancies but patients may eventually relapse. Here, the authors show that CD19 CAR-T resistance in pre-B cell ALL can be due to the induction of a myeloid lineage switch through an epigenetic alterations in master regulators of B cell development.

## Graphical abstract

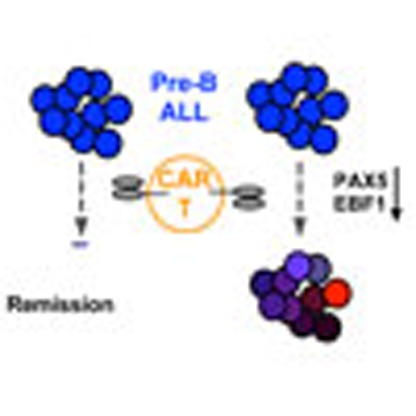

Acute leukaemia is a heterogeneous group of clonal malignancies, classified as lymphoblastic (ALL), myeloid (AML) or mixed phenotype (MPAL)^1^. These subtypes have distinct molecular and genetic alterations that affect prognosis, guide treatment^2^,^3^,^4^, and are found in pre-leukaemic clones^5^,^6^,^7^. Nonetheless, lineage switch has been reported as a rare phenomenon, typically associated with poor prognosis^8^,^9^,^10^,^11^, with myeloid leukaemia relapsing as lymphoid (T or B) lineage and vice versa^8^,^9^,^10^,^11^,^12^,^13^,^14^,^15^,^16^,^17^,^18^,^19^. This process occurs during or following chemotherapy, and may represent selection of an undetected clone in the original leukaemia or reprograming^20^. Lineage switching occurs more often with specific genetic subtypes of leukaemia such as MLL-rearranged that may have greater inherent plasticity.

Use of adoptively transferred T cells armed with chimeric antigen receptors (CAR-T) is a promising new cancer therapy^21^,^22^,^23^. CARs are artificial receptors containing an extracellular recognition domain (usually an antibody-derived single-chain fragment variable region) combined with signalling domains, typically CD3-zeta plus a costimulatory domain from CD28, CD137, OX40 or others^21^. CAR-T targeting CD19 have generated high complete remission rates in a variety of B-cell malignancies^24^,^25^,^26^,^27^,^28^,^29^, most notably refractory or relapsed ALL^26^,^27^,^28^. Despite these promising results, relapse attributable to T-cell failure^27^ or tumour antigen loss^28^,^30^ may limit the effectiveness of CAR-T. CD19 is essential to B-lineage development^31^,^32^, thus antigen loss was an unexpected form of escape from CAR-T and was recently found to be explained in some cases by alternative splicing of CD19 lacking the CAR-binding epitope but with retention of a functional protein^30^.

Analysis of the impact of CAR-T on leukaemia *in vivo* has been limited in xenograft models due to lethal xenogeneic graft-versus-host disease precluding long-term studies. Furthermore, lack of an intact host immune system could impact *in vivo* behaviour of both CAR-T cells and leukaemia. To overcome these limitations and to study leukaemia resistance in the setting of CD19 CAR pressure, we used *in vivo* murine ALL models in which initial clearance of leukaemia by CD19 CAR-T cells is achieved with long-term persistence of CAR-T. Here we describe lineage switch as a mechanism of CD19 CAR-T resistance. Using genomic analysis of myeloid lineage switched leukaemias generated under CD19 CAR pressure and gene-editing techniques, we show this phenomenon is not simply due to alterations of CD19 but rather from a global reprograming of ALL with inherent lineage plasticity.

## Results

### ALL blast phenotypic alterations in patients post-CD19 CAR

**Figure 1:**
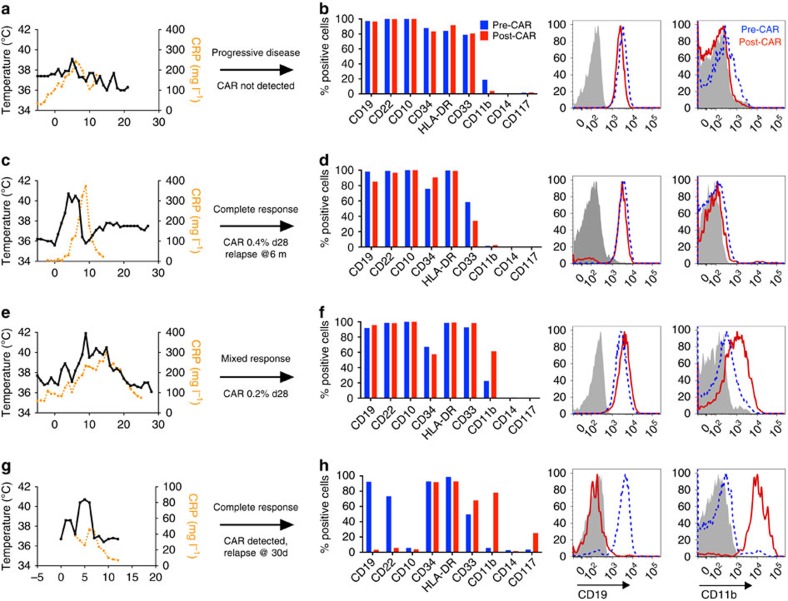
Phenotypic alterations in clinical ALL samples following CD19 CAR. (**a**,**c**,**e**,**g**) Fever curves, CRP values and clinical response to CD19 CAR treatment. (**b**,**d**,**f**,**h**) Bar graph demonstrating percent of cells expressing cell surface markers by flow cytometry in the bone marrow, gated on leukaemic blasts, with representative flow cytometry histograms of CD19 and CD11b on the right (grey, control; blue, pre-CAR; red, post CAR). (**a**,**b**) Pre-CAR sample and day +30 post-CAR sample from a patient who did not experience CRS following CD19 CAR. (**c**,**d**) Pre-CAR and +180 days post-CAR samples from a patient with normal karyotype multiple relapsed ALL, who had a severe CRS followed by an MRD-negative complete response with CD19 CAR, with no CAR^+^ cells persisting beyond 60 days. (**e**,**f**) Pre-CAR and day +30 post-CAR samples from a patient with normal karyotype ALL who experienced a mild CRS and CAR expansion, but had persistent disease. (**g**,**h**) Pre-CAR and post-CAR samples from an infant with MLL-rearranged ALL, treated with CD19-41BB-zeta CAR, who relapsed with myeloid blasts.

Results from our trial CD19-CAR containing a CD28 costimulatory domain were previously reported, demonstrating excellent remission rates but relatively short *in vivo* persistence^27^. We report results and leukaemic phenotype of three patients treated on this trial who were either resistant to therapy or experienced a subsequent relapse. Patient ALL_H0112 did not develop cytokine release syndrome (CRS) or CAR expansion, with persistent leukaemia on day 30 that retained an identical cell surface phenotype to pre-CAR leukaemia (Fig. 1a,b). Patient ALL_H0082 experienced severe CRS requiring tocilizumab and steroids, followed by a complete remission. He had persistent CD19 CAR T cells on day 30 (0.4% of peripheral blood), with a subsequent relapse 6 months following CAR therapy with no detectable CAR at that point (Fig. 1c,d). On initial relapse, a small population of blasts lacking only CD19 was identified with an otherwise unchanged leukaemic phenotype (Fig. 1d and Supplementary Fig. 1). Surprisingly, we could not identify CD19 splicing events as the cause, as recently reported^30^, suggesting other mechanism involved. Patient ALL_H0118 was treated for a normal-karyotype, multiply relapsed ALL and experienced mild CRS with transient *in vivo* CAR-T expansion, but persistent leukaemia at day 28 despite presence of CD19 CAR T cells (0.2% in peripheral blood). Under transient CAR pressure post-CAR leukaemia demonstrated stable expression of CD19 and increased CD11b compared to pre-CAR leukaemia (Fig. 1e,f). Finally, complete remission induced in an infant with MLL-rearranged ALL treated with a CD19 CAR containing a 41BB costimulatory domain with CAR persistence showed initial clearance of blasts with a relapse at day 30 with leukaemia that lost of B-cell markers, retained CD34 and HLA-DR, and gained of CD11b and CD33 expression (patient ALL_H0140, Fig. 1g,h). Thus, CD19 CAR-T can induce varying degrees of phenotypic change in patients with pre-B cell ALL including change to a myeloid phenotype.

### CD19 CAR induces myeloid lineage switch of murine ALL

**Figure 2:**
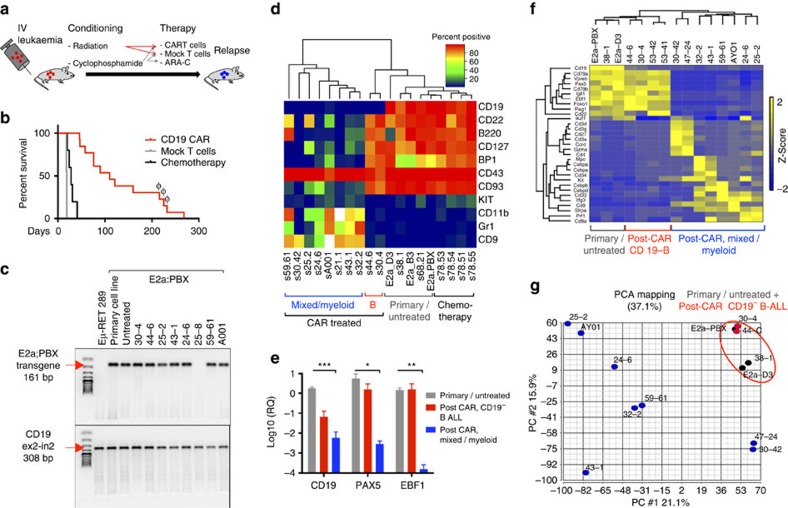
Distinct phenotypic and genomic alterations in pre-B cell induced by CD19 CAR pressure *in vivo*. (**a**) Schematic design of *in vivo* murine experiments: mice were injected with leukaemia, followed by lymphodepletion (5 Gy radiation or 4 mg cyclophosphamide) and adoptive T-cell therapy (CAR/Mock T cells) or chemotherapy (4 mg per mouse cyclophosphamide on day 4 followed by 2.5 mg ARAC on days 5 and 10). (**b**) Survival curve of mice treated with chemotherapy (black, *n*=5), mock T cells (blue, *n*=9) or CD19 CAR T cells (red, *n*=13). Samples marked with φ had no E2a:PBX transgene on PCR and were not further analysed. (**c**) PCR for E2a:PBX transgene of Eμ-RET cells (negative control), E2A:PBX parental cell line, and splenocytes harvested from leukaemic mice treated with CD19 CAR. CD19 gene (exon2–intron2) was used as control for DNA quantity. (**d**) Primary and post-CAR relapse leukaemia was analysed by multicolour flow cytometry. Heatmap representing percent of positive cells for multiple lineage markers for each sample. Unsupervised hierarchical clustering is shown above. Mouse IDs as in Supplementary Table 1 are reported below. (**e**) Quantitative RT PCR for CD19, PAX5 and EBF1 mRNA in primary E2a:PBX leukaemia or mock-treated relapse (*n*=7, grey), post-CAR CD19^−^ B-ALL relapses (*n*=2, red) and post-CAR mixed/myeloid relapses (*n*=7, blue). Data represented as fold change in gene expression over GAPDH, error bars represent s.e.m. *P<0.05, ^**^P<0.01, ^***^P<0.001 (**f**) Heatmap demonstrating changes in gene expression of selected hematopoietic transcripts measure by RNA sequencing. Unsupervised clustering is shown above. (**g**) Principal component analysis of RNA sequencing data from primary and post-CAR samples. Red circle indicates clustering of primary and early-post-CAR samples.

Next we tested the impact of CD19 CAR-T in two murine ALL models. The E2a:PBX murine B-lineage ALL line was propagated from splenocytes of an *E2a:PBX1* (also known as *TCF3:PBX1*) transgenic mouse crossed to a CD3^−/−^ mouse with spontaneous pre-B ALL development^33^. This aggressive E2a:PBX cell line maintained stable pre-B cell phenotype during multiple passages *in vitro* and *in vivo*^34^,^35^, and results in lethality of immunocompetent C57Bl/6 mice within 21 days despite treatment with chemotherapy (Fig. 2a,b). A second generation murine CD19 CAR with a CD28 costimulatory domain^36^ was retrovirally transduced into syngeneic murine T cells and administered to mice bearing E2a:PBX leukaemia following lymphodepletion (radiation or cyclophosphamide), resulting in long-term remissions with persistence of functional CAR-T cells (Supplementary Fig. 2). Despite the early potency, all CAR-T recipients succumbed within 1 year from progressive leukaemia (Fig. 2b), with maintained expression of the E2a:PBX transgene in the majority of post-CAR relapses (Fig. 2c), confirming derivation from the original leukaemia. In contrast to mock-treated or chemotherapy-treated mice that relapsed with a pre-B ALL phenotype identical to the pre-injected cells, all post-CAR leukaemic relapses lost CD19 expression. Further characterization of post-CAR leukaemia demonstrated that earlier relapses maintained pre-B phenotype with isolated CD19 loss, whereas later relapses acquired multiple phenotypic changes including loss of additional B-cell markers (Fig. 2d).

Resistance to CD19 CAR in patients can result from alternative splicing of exon 2 of *Cd19* resulting in lack of expression of the targeted epitope and cytosolic localization^30^. Using *Cd19* exon-specific primers, we identified a similar mechanism for early post-CAR CD19-negative relapse of E2a:PBX with isolated loss of an exon 1–3 junctional transcript indicating lack of exon 2 expression but maintained expression of other exons (Supplementary Fig. 3). In contrast, later relapses did not express any *Cd19* transcripts based on PCR and RNA sequencing. Furthermore, absence of *Cd19* transcripts in late post-CAR relapses was accompanied by reduced expression of *Pax5* and *Ebf1* (Fig. 2e), important B-cell regulatory transcription factors^37^,^38^ consistent with lack of the B-cell developmental program. Decreased expression of *Pax5* and *Ebf1* also occurred in the post-CD19 CAR leukaemia from patient ALL_H0140 demonstrating lineage (Supplementary Fig. 4). No genetic loss of *Pax5* was identified in the relapsed samples with low *Pax5* expression in both murine and human samples post CAR, suggesting upstream and/or epigenetic regulation. Loss of *Pax5* or *Ebf1* has been associated with lineage switch in human non-malignant B cells and pre-B ALL^38^,^39^,^40^. Similar to the patient presented in Fig. 1g,h, extended phenotyping using multi-parameter flow cytometry and identified that, in addition to loss of B-cell markers, late relapses acquired phenotypic markers of alternative lineages, including CD11b, Gr1 and cKIT (Fig. 2d and Supplementary Table 1). Unsupervised hierarchical clustering of cell-surface markers further illustrated that early relapses were similar to parental pre-B ALL with the exception of CD19 loss, whereas later relapses were characterized by multiple phenotypic alterations (Fig. 2d). Unsupervised clustering of RNA sequencing data further confirmed distinct features of later compared with earlier relapses (Fig. 2f and Supplementary Fig. 5), with loss of B-cell-associated transcripts and gain of expression of myeloid or T-cell genes consistent with lineage switching. Interestingly, principal component analysis revealed greater diversity among lineage switch relapses (Fig. 2g), indicating that persistent CD19 CAR can induce multiple gene expression profiles that allow pre-B ALL escape.

We next used an Eμ-RET murine pre-B ALL to test the hypothesis that CAR resistance mechanism may differ depending on the genetic basis^41^,^42^. As with the E2a:PBX1 model, CD19-CAR treatment induced prolonged remissions in Eμ-RET leukaemia with occasional late mortality (Supplementary Fig. 6). However, at necropsy bone marrow showed normal-appearing hematopoiesis with no evidence of leukaemia by flow cytometry, PCR for the Eμ-RET transgene and failed to generate leukaemia following injection into secondary recipients (Supplementary Fig. 6). Persistence of CAR T cells was confirmed by PCR. Thus, in contrast to E2a:PBX leukaemia, Eμ-RET pre-B cell ALL did not relapse following CD19 CAR via lineage switch suggesting that the occurrence of this resistance mechanism may depend on the genetic driver.

### Myeloid clones not detectable prior to CD19 CAR exposure

**Figure 3:**
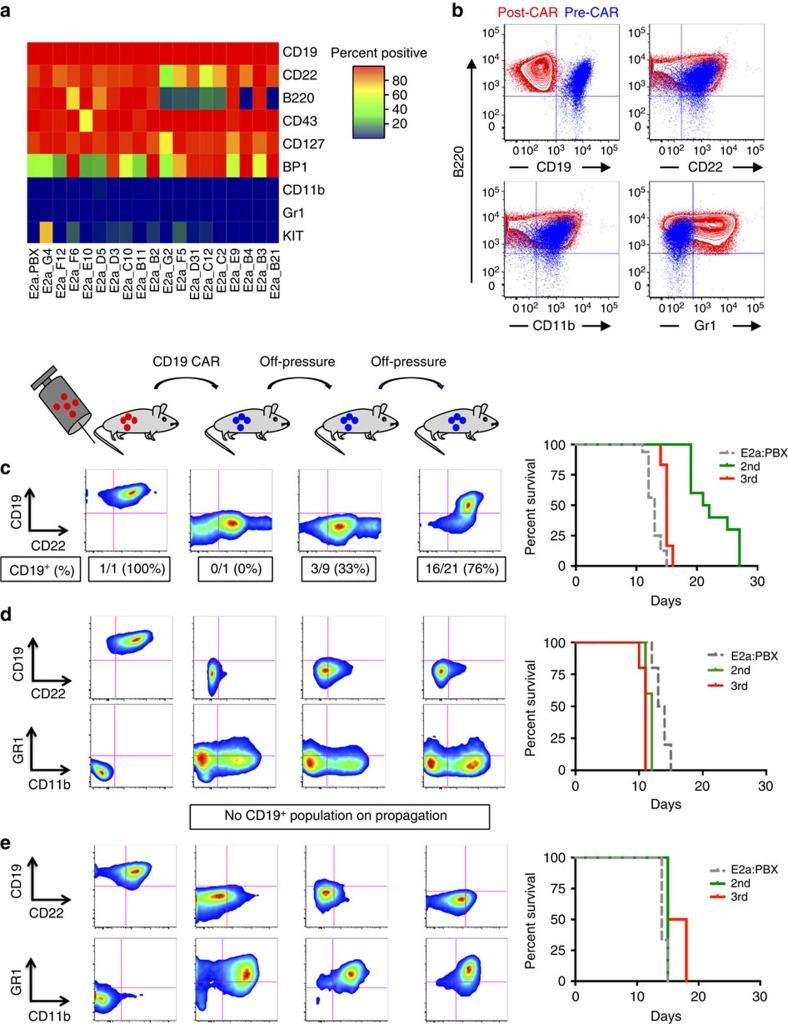
Lineage switch phenotype is not detectable in parental leukaemia and is stable when CAR pressure is removed. (**a**) Primary E2a:PBX ALL was single-cell cloned by limiting dilution, expanded and analysed by flow cytometry. Heatmap of cell surface marker expression evaluated by flow cytometry. (**b**) CD45.2^+^ B220^+^ leukaemic blasts from sample 59–61 obtained at day 58 following CAR demonstrating intermediate B/myeloid phenotype expressing for CD22, CD11b and Gr1, but negative for CD19. (**c**–**e**) *In vivo* passage of post-CAR CD19^−^ relapsed leukaemia, with E2a:PBX control, in absence of additional CAR treatment. (**c**) B/Myeloid biphenotypic sample 25–2 passaged twice off pressure, with re-emergence of a CD19^+^ population in 16/21 mice in 3rd passage (E2a:PBX control *n*=16, 2nd *n*=10, 3rd *n*=12). (**d**) Lineage switch samples 24–6 (*n*=5 per group) and (**e**) A001 (E2a:PBX control *n*=3, 2nd *n*=3, 3rd *n*=4) passaged twice. Respective survival curves for 1 of 2 experiments shown on the right.

Lineage switch in patients following cytotoxic therapy results from selection or genetic reprogramming^20^. The diverse gene expression profile of the post-CAR mixed phenotype and myeloid relapses derived from E2a:PBX1 ALL following CD19 CAR-T suggested emergence of leukaemia with distinct lineage characteristics resulted from reprogramming. To evaluate for the possibility of selection of a pre-existent alternative lineage cell we performed single cell cloning of E2a:PBX pre-B ALL cells. All single-cell subclones expressed pre-B-cell markers CD19, CD22, CD127 and CD43, with some variation in the expression of B220, BP1 and CD117 (c-kit) indicating that, although phenotypic subclones exist, none display the full phenotypes of the emergent post-CAR relapses (Fig. 3a). One subclone rapidly lost CD117 expression in culture and following *in vivo* passage (Supplementary Fig. 7) suggesting inherent instability of this phenotype. Next, we performed antibody-based magnetic depletion to select for CD19-negative cells. None of the cultures derived from sorted E2a:PBX cells expressed myeloid-associated markers (Supplementary Fig. 8), suggesting that myeloid-subclones of E2a:PBX ALL are absent or the frequency was below the level of detection of these assays. Finally, some post-CAR relapses co-expressed both myeloid and lymphoid makers, demonstrating a mixed B/myeloid phenotype as a potential intermediate in the switching process (Figs 2d and 3b). Taken together these results indicate that emergence of a leukaemic phenotype with distinct lineage characteristics from the pre-CAR ALL likely results from reprogramming of a cell with lineage plasticity during *in vivo* CD19 CAR immunopressure.

### Phenotypic stability of post-CAR lineage switched leukaemia

To study the phenotypic stability of the post-CAR relapses, we serially passaged CD19-negative E2a:PBX leukaemic cells into secondary and tertiary recipients (Fig. 3c) without CAR-T cells. Post-CAR relapse leukaemia caused mortality in secondary recipients within 30 days. CD19-negative, B-phenotype post-CAR relapses (samples 30–4 and 44–6) showed persistent lack of CD19 expression following transfer, with a stable pre-B phenotype (Supplementary Fig. 9). Isolated CD19-negative relapses from patients following CD19-targeted immunotherapy^30^ are similarly stable when passaged in immunodeficient mice (Supplementary Fig. 9). In contrast, intermediate phenotype, mixed B/myeloid post-CAR leukaemia, with complete loss of CD19 gene expression but residual expression of B-cell markers (CD22 or B220), regain of CD19 occurred in some secondary and the majority of tertiary recipients suggestive of a persistent pre-B cell developmental program despite partial phenotypic change (Fig. 3c). Interestingly, post-CAR relapses with loss of all B-lineage surface markers and gene expression profile consistent with complete lineage switch, were phenotypically stable on serial passaging (Fig. 3d,e). Overall, this provides evidence that the ability for pre-B ALL to switch lineages under CAR immune pressure can result in emergence of a stable, alternatively differentiated CAR-resistant phenotype.

### Genomic accessibility associates with lineage of relapse

**Figure 4:**
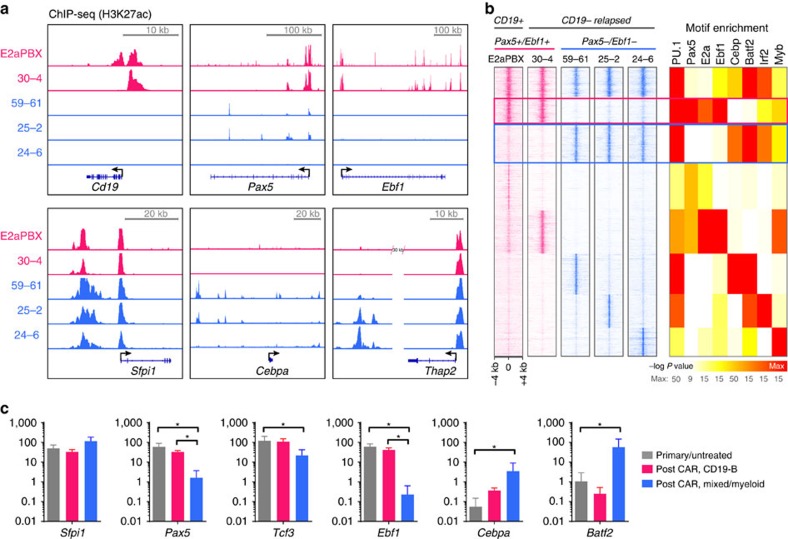
Epigenomic signature demonstrates switch of lineage-specific active chromatin. ChIP-Seq of H3K27Ac was performed in original disease (E2a:PBX) and four post-CD19-CAR E2a:PBX relapses: a lymphoid CD19-B-ALL (30–4), two mixed B/myeloid samples (5,961 and 252) and a myeloid sample (24–6). (**a**) ChIP-seq tracks of H3K27ac signal at promoter and enhancer regions for *Cd19*, *Pax5*, *Ebf1*, *Sfpi1* (encoding PU.1), *Cebpa* and *Thap2* for the four samples (lymphoid marked in red, myeloid in blue). (**b**) Transcription factor motif analysis of shared and lineage-specific enhancers was performed, with a more significant *P* value calculated for more abundant motifs within H3K27Ac-bound chromatin. The Pax5, E2a and Ebf1 motifs were found in regions lost on lineage switch (red box), and absent from newly activated chromatin in samples 59–61, 25–2 and 24–6 (blue box). (**c**) mRNA expression in fragments per kilobase of transcript per million mapped reads (FPKM) of the transcription factors with enriched motifs (shown in Fig. 4b), grouped according to clustering in Fig. 2d and Supplementary Fig. 5. Error bars represent s.d. *P<0.05.

We next performed chromatin immunoprecipitation sequencing of the H3K27ac histone mark in lymphoid, mixed B/myeloid and myeloid relapses to evaluate the changes in activated chromatin sites in lineage-specific transcriptional as a potential mechanism of post-CAR lineage reprogramming. In correlation with the transcriptome profile (Fig. 2f), the post-CAR CD19 negative lymphoid relapse had H3K27ac signals in B-cell-associated transcription factor promoter regions including *Cd19*, *Pax5* and *Ebf1* (Fig. 4a). In contrast, mixed or myeloid relapses lost H3K27ac marks at these sites, with gain of H3K27ac in myeloid enhancers such as the +37 kb *Cebpa* enhancer and the *Thap2* enhancer. All lineages retained the −12 kb *Sfpi1* enhancer driving PU.1 (Fig. 4a), a master transcription factor associated with myeloid lineage^43^,^44^. Moreover, using genome-wide analysis of lineage-specific enhancers, *Ebf1* and *Pax5* motif enrichment in chromatin sites was present only in the original leukaemia and CD19^−^ B phenotype relapse (30–4), but were essentially absent in myeloid or mixed B/myeloid samples (Fig. 4b) which were, instead, characterized by a gain in regions enriched in motifs for *Cebp, Batf2* and *Irf2*, significant myeloid transcription factors, with correlating mRNA expression (Fig. 4c). Thus, lineage switching is associated with changes in the genomic accessibility of factors driving lineage differentiation.

### Genomic editing can recapitulate lineage reprograming

**Figure 5:**
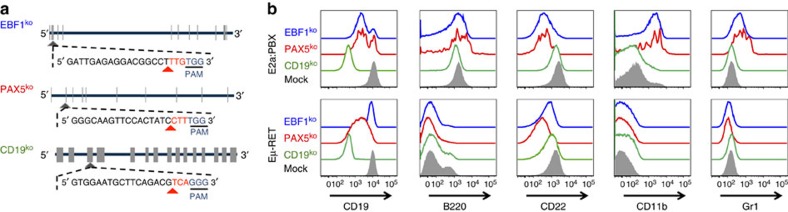
Knockout of *Pax5* or *Ebf1* in E2a:PBX pre-B-cell ALL results in a phenotype mirroring relapse under CAR pressure. (**a**) Representative structure of *Cd19*, *Pax5* and *Ebf1*, exons marked in tabs. Guide-RNA design for each knockout shown, with the CAS9 PAM site underlined, and cleavage site marked with a red arrowhead. (**b**) Flow cytometry plots for murine leukaemia cell lines E2a:PBX (upper panel) and Eμ-RET 309 (bottom panel) following CRISPR/CAS9 knockout of CD19 (green), PAX5 (red), EBF1 (blue) or mock (grey shadow).

In non-malignant B cells and pre-B cell leukaemia, PAX5 and EBF1 are the primary transcription factors determining the B-cell developmental program^38^,^39^,^40^,^45^,^46^,^47^,^48^ and regulating expression of CD19, a co-receptor for pre-BCR signalling^31^. Thus, we hypothesized that absence of CD19 would be insufficient to drive complete lineage switch phenotype in E2a:PBX ALL whereas deletion of PAX5 or EBF1 would result in similar phenotypes to those occurring in late post-CAR relapses. Using the CRISPR/Cas9 system, we ablated the *Cd19*, *Pax5* or *Ebf1* from E2a:PBX and Eμ-RET ALL (Fig. 5a). Indeed, *Cd19*-deficient E2a:PBX leukaemia retained a stable pre-B lineage in culture and *in vivo* and comparable lethality to the parental E2a:PBX. As expected, these cells demonstrated inherent resistance to CD19 CAR maintaining a lymphoid phenotype at post-CAR relapse (Supplementary Fig. 10). In contrast, both *Pax5* and *Ebf1*-deficient E2a:PBX expressed CD11b and Gr1 (Fig. 5b). Eμ-RET ALL did not upregulate myeloid antigens following *Ebf1* or *Pax5* deletion consistent with the inability to lineage switch under CD19 CAR pressure *in vivo*. Thus, lineage switch following pressure on the B-cell lineage by CD19 CAR can be recapitulated through ablation of master regulators of B-cell development but only in ALL with the capacity to utilize this mechanism for resistance to CD19-targeted immunotherapy.

## Discussion

We demonstrate that persistent CD19 CAR-T-cell immune pressure can induce lineage switch as a mechanism of CAR resistance. This can occur in patients following CD19 CAR and can be recapitulated in a syngeneic murine model in which pre B cell ALL is driven by the clinically relevant E2a:PBX1 transgene allowing comprehensive study. Importantly, we demonstrate that immune pressure against CD19 by CAR-T cells can result either in emergence of a rapidly relapsing leukaemia that has selectively lost the CD19 CAR epitope, as reported in patients following CD19 CAR^30^, or a lineage-switched leukaemia that results from reprogramming. We show that the deletion of CD19 alone is insufficient to replicate the lineage switch mechanism occurring under CD19 CAR pressure *in vivo* but rather requires lineage reprogramming induced by ablation of the B-cell transcription factors resulting in complete loss of B-cell developmental pathways. Failure for CD19 deletion to recapitulate lineage switch would be predicted as CD19 is regulated downstream of the B-cell program^31^,^38^. Interestingly, a similar process of plasticity-driven evasion of lineage-specific immunotherapy following CD19-directed immunotherapy has recently been described following CD19-CAR in CLL with Richter transformation into a plasmablastic lymphoma^49^ and in two patients with MLL rearranged ALL transformed to AML^50^, and following bispecific CD3-CD19 antibody in ALL^51^.

When attempting to model human leukaemia in mice by introducing pre-B-ALL-associated transgenes (such as *ETV6:RUNX1*, *E2a:HLF* or *MLL:AF4*) into murine hematopoietic cells, the resultant leukaemic phenotype can vary and often may emerge as T or myeloid leukaemia^52^. The E2a:PBX leukaemia model used in this work was based on an *E2a:PBX* x CD3^−/−^ transgenic mouse^33^, that has the propensity to spontaneously develop B, myeloid or B/myeloid leukaemia. The experiments performed here utilized an E2a:PBX ALL cell line from this murine model that is fully and stably a pre-B-cell phenotype. Nonetheless, the inherent potential for the E2a:PBX transgene to drive both lymphoid and myeloid leukaemia is analogous to MLL rearranged pre-B ALL in humans, the subtype associated with the post-CAR lineage switch described here. Ongoing deletion of all B-lymphoid programmed leukaemia through CD19 immune pressure or genetic ablation reveals the myeloid potential. This is in contrast to the Eμ-RET-driven leukaemia, that is driven by an Eμ-enhancer and lacks a myeloid potential in several publications^41^,^53^,^54^, and which retains stable B lineage commitment even with *in vivo* CD19-CAR immune pressure or *in vitro* deletion of *Ebf1* and *Pax5*.

Indeed, the plasticity of pre-B cells and pre-B ALL has been reported in multiple models, most of which involve specific genetic alterations such as deletion of PAX5 that has been shown to enable B cells to reprogram into macrophages or functional T cells^40^,^46^,^47^,^48^. Similarly, following knockout of EBF1, committed pro-B cells differentiate into T cells when transferred into immunodeficient mice^55^. PAX5 haploinsufficiency is a common genetic feature in ALL, which may result in either CD19^+^ or CD19^−^ leukaemia^56^. In our model, persistent CAR T cells targeted specifically to the B-cell co-receptor, CD19, led to reemergence of a lineage switch similar to models of PAX5 or EBF1 knockout. Importantly, CD19 loss itself was not sufficient to drive the observed lineage switch. Rather, CD19-expressing B-ALL cells are continuously being deleted under this pressure, until leukaemia either downregulates CD19 (in two of our samples) or reprograms via downregulation of PAX5 and EBF1 to drive a CD19 CAR-resistant relapse. Interestingly, in a recent conditional knock-in model of E2a:PBX leukaemia, initiation by this transgene occurred almost exclusively when introduced in hematopoietic stem cells or early B cells prior to CD19 suggesting that the transformation event occurs in a less-differentiated cell likely to have pluripotentiality^57^. Altogether, this suggests that the inherent lineage plasticity of the original leukaemic cell will determine the potential to reprogram, under lineage-specific pressure.

Current attempts to overcome the limitations of targeted-immunotherapy are primarily focused on persistence and specificity of the effector cells. While this is undoubtedly critical to maximize the potential of adoptive cell therapy, we suggest that understanding the full spectrum of the mechanisms by which leukaemia can escape will also be important. A diversity of specific genetic translocations is one of the hallmarks of pre-B ALL in humans, resulting in the ability to guide therapy, predict prognosis and develop molecularly targeted strategies. Our findings expose important aspects of pre-B-cell ALL biology that could impact selection of immunotherapeutic targets. Indeed, we describe lineage changes following CD19 CAR in patients, including lineage switch in an MLL-rearranged patient. As ALL can harbour translocations seen in both lymphoid and myeloid lineages^10^,^18^,^19^,^58^,^59^, and the presumed cell of origin originated in the hematopoietic stem cell compartment^5^,^60^,^61^,^62^, the ability to reprogram may create additional opportunities to escape pressure against single antigens. Targeting multiple antigens or therapy that focuses on a leukaemia-initiating cell may represent a strategy to prevent this occurrence. It is interesting to note that CD22 is maintained on the intermediate phenotype relapses suggesting that simultaneous pressure on CD19 and C22 might be strategy to reduce the likelihood of lineage switching. These results have important implications for optimizing adoptive cell therapy for ALL and indicate that, at least for some subtypes of pre-B-cell ALL, methods to prevent lineage switch may facilitate the important goal of durable remissions following targeted immunotherapy for ALL.

## Methods

### Patient samples

Patients with relapsed or refractory leukaemia were enroled on a clinical trial utilizing autologous CD19-chimeric antigen receptor T cells with a CD28 costimulatory domain on a retroviral backbone (NCT01593696) previously reported^27^ and on a trial with a 41BB-costimulatory domain on a lentiviral backbone (NCT02028455). Informed consent was obtained from all participants. Bone marrow specimens were collected for research and de-identified according to an institutional IRB-approved protocol.

### Tumour cell lines

A murine pre-B ALL carrying the human E2a:PBX transgene crossed to a CD3ɛ^−/−^ was generously provided by Dr Janetta Bijl. These cells were developed into a stable cell line expressing pre-B markers. Leukaemia cells were cultured in 10% complete mouse media (CMM) containing RPMI 1,640 with 10% heat-inactivated fetal calf serum, 1% glutamin, 1% HEPES, 1% nonessential amino acids, 1% sodium pyruvate, 1% penicillin/streptomycin, (Invitrogen) and 50 μM 2-mercaptoethanol. Eμ-RET cell lines 289 and 309 were provided by Dr Alix Seif^42^, and grown in 20% CMM. Human cell lines NALM6 (ALL, DSMZ ACC-128) and MOLM13 (AML, DSMZ ACC-554) were used as controls for human experiments. All cell lines were tested and found negative for mycoplasma contamination. Leukaemia was injected IV by tail vein in all experiments. Single cell clones, when mentioned, were generated by limiting dilution with final concentration of 30 cells per 10 ml, divided into 96-well plates.

### Murine CAR production

Murine CD19 CAR construct with a CD28 costimulatory domain on an MSGV retroviral backbone was provided by Dr James Kochenderfer^36^. For murine CAR T-cell production^35^, splenocytes were harvested, filtered via 70-μm restrainer, red-blood cell depleted using ACK-lysis buffer (Lonza), and T-cell enriched using CD3+ T-cell enrichment column (R&D). Enriched product was incubated in CMM and activated with Mouse T-Activator CD3/CD28 Dynabeads (Life Technologies) in the presence of IL2 (30 U ml^−1^) and IL7 (10 ng ml^−1^). On days 2 and 3 of culture, retronectin-coated plates were spun for 2 h with retroviral supernatant, followed by incubation of the T-cell-enriched product for 24 h. On day 4 of culture, T cells were removed from retronectin-coated plates and from activating beads, and expanded for an additional 24–36 h in the presence of the aforementioned cytokines.

### Murine CAR treatment protocol

C57BL/6 (H-2^b^ CD45.2^+^), B6-Ly5.2 (H-2^b^ CD45.1^+^) and Balb/C (H-2^d^, CD45.2^+^) mice were purchased from the National Cancer Institute (NCI) animal production program. Mice were used between 6 and 10 weeks of age. All animals were kept in a pathogen-free facility, and were treated under protocols approved by the Animal Care and Use Committee at the National Cancer Institute. Overall, mice were injected with leukaemia on day 0, conditioned with radiation (500 cGy) on day 4, followed by adoptively transfer of CD19-CAR or mock T cells on day 5. Alternatively, conditioning of mice included cyclophosphamide (4 mg per mouse) instead of radiation, prior to adoptive cell therapy or additional chemotherapy, which included two doses of ARA-C (2.5 mg per dose per mouse) on days 5 and 10. All mice were monitored three times a week for survival, and were killed when paralysed or moribund, and bone marrow and spleen were harvested for analysis.

### Antibodies and flow cytometry

The following conjugated anti-murine antibodies were used for flow cytometry: FITC-anti-CD45.1 (A20, #11-0453), FITC-anti-CD9 (MZ3, #11-0091), PE-Cy7-anti-CD45.2 (104, #12-0454), PE-anti-CD127 (A7R34, #12–1,271), efluor450-anti-CD19 (eBio1D3, #48-0193), efluor-anti-CD45.2 (104, #48-0454), PECy7-anti-CD117 (2B8, #25–1,171), efluor-anti-CD34 (RAM34, #50-0341; eBioscience); APC-Cy7–anti-B220 (RA3-6B2, #103224), APC-anti-CD22 (OX-97, #126110), PE-Cy7-anti-CD93 (AA4.1, 136506; Biolegend); PE-anti-CD22 (Cy34.1, #553384), APC-anti-CD11b (M1/70, #553312), PE-anti-BP1 (BP-1, #553735), FITC-anti-GR1 (RB6-8C5, #553127; BD Biosciences). CAR detection was performed using protein-L with PE-Streptavidin (BD Biosciences). The following conjugated anti-human antibodies were used: efluor45-anti-CD19 (HIB19, #48-0199), FITC-anti-CD19 (HIB19, #11-0199)), efluor-anti-CD34 (4H11, #48-0349), PE-Cy7-anti-CD11b (ICRF44, #25-0118), FITC-anti-CD14 (61D3, #11-0149), APC-anti-CD117 (104D2, #17–1,178), PE-anti-CD33 (WM-53, #12-0338), PerCP-Cy5.5-anti-CD45 (HI30, #45-0459; eBioscience); PE/Cy7-anti-CD10 (H110a, #312214, Biolegend); PE-anti-CD22 (S-HCL1, #347577) and PE-anti-HLA-DR (G46-6 #556644; BD Biosciences). Samples were analysed on a BD LSR-Fortessa (BD Biosciences), data collected using FACS Diva software and analysed using FLowJo version 9.6.4 (Treestar).

### Nucleic acid extraction, PCR and RNA sequencing

Nucleic acid extraction was performed using Qiagene AllPrep kit. PCR primers for DNA and cDNA are reported in Supplementary Table 2. PolyA-selected RNA libraries were prepared for RNA sequencing on the Illumina NextSeq platform using TruSeq chemistry according to the manufacturer's protocol. 150 bases long paired-end reads were assessed for quality and reads were mapped using CASAVA (Illumina). The generated fastq files were used as input for mapping using TopHat2 (ref). Cufflinks (http://cufflinks.cbcb.umd.edu/) was used to assemble and estimate the relative abundances of transcripts mapped with TopHat2 at both the gene and transcript level (FPKM). FPKM values were log2 transformed and Z-scored.

### Chromatin immunoprecipitation sequencing

Chromatin was prepared from 15 × 10^6^–20 × 10^6^ cells using the ChIP-IT High Sensitivity Kit as per the manufacturer's protocol (Active Motif). Shearing occurred under the following conditions using the EpiShear Probe Sonicator-Amplitude: 0.3/Pulse: 30 s ON, 30 s OFF/Cycles: 56. Antibodies for Histone H3K27ac (Active Motif) were used for the immunoprecipitation reactions at 10 μg per reaction. Library preparation and sequencing utilized the TruSeq ChIP Sample Prep Kit and NextSeq 500 High Output Kit (75 cycles) according to the manufacturer's instruction (Illumina). The ChIP-IT qPCR Analysis Kit validated results using primers for CD19 and PAX5 (Active Motif).

### ChIP-seq enhancer and motif analysis

H3K27ac peaks >2,500 bp from the nearest TSS in mouse genome build mm9 were classified as enhancers. Using Bedtools intersect, we identified peaks found in naive and relapsed cell types that were classified as either lineage un-specific, and *Pax5*+/*Ebf1*+ specific, *Pax5*−/*Ebf1*− specific, or single lineage-specific clusters. Each set of enhancer peaks were analysed for motif enrichment with the HOMER package scanning sequence for known motifs within 500 bp of either side of the peak centre.

### Lentiviral CRISPR design and transduction

Guide-RNAs (Supplementary Table 2) were optimized by http://crispr.mit.edu/, cloned into LentiCRISPR v2 plasmid (Addgene Plasmid 52,961), and finally transformed as previously published. Plasmids were co-transfected with packaging plasmids RRE, pMD-G and REV were transfected into HEK293T cells. After 2 days, CRISPR supernatants were harvested and filtered through a 0.45 μm low protein binding membrane (Millipore) and concentrated using Lenti-X concentrator (Clontech), resuspended in PBS and used immediately or stored at −80 °C. For viral transduction, 10^5^ leukaemia cells were incubated with 10 μl of concentrated viral supernatant for 2 days, followed by expansion in CMM. Cell phenotype was assessed by flow cytometry, followed by sorting of cells with phenotypic alterations and single-cell cloning. Sequencing was performed on single-cell clones to confirm genotypic alterations.

### Statistical analysis

Survival of mice was analysed through the Kaplan–Meier method, using Wilcoxon rank test. For continuous variables, the Mann–Whitney method was used to compare sample groups (*n*<30 per group). Statistical analysis was performed using GraphPad Prism version 6 for Macintosh (Graphpad Software). *P* values <0.05 were considered significant.

### Data availability

The data reported in this study have been deposited in the Gene Expression Omnibus (GEO) database, www.ncbi.nlm.nih.gov/geo (accession numbers GSE83205 and GSE83203).

## Additional information

**How to cite this article:** Jacoby, E. *et al.* CD19 CAR immune pressure induces B-precursor acute lymphoblastic leukaemia lineage switch exposing inherent leukaemic plasticity. *Nat. Commun.* 7:12320 doi: 10.1038/ncomms12320 (2016).

## Supplementary Material

Supplementary Information Supplementary Figures 1-10 and Supplementary Tables 1 & 2.

Peer Review File
