# Supplementary material for: CD19 CAR immune pressure induces B-precursor acute lymphoblastic leukaemia lineage switch exposing inherent leukaemic plasticity
Source: Nat Commun. 2016 Jul 27;7:12320. doi: 10.1038/ncomms12320 (PMC4974466; doi:10.1038/ncomms12320)
Supplement: Supplementary Information — Supplementary Figures 1-10 and Supplementary Tables 1 & 2. [file ncomms12320-s1.pdf]

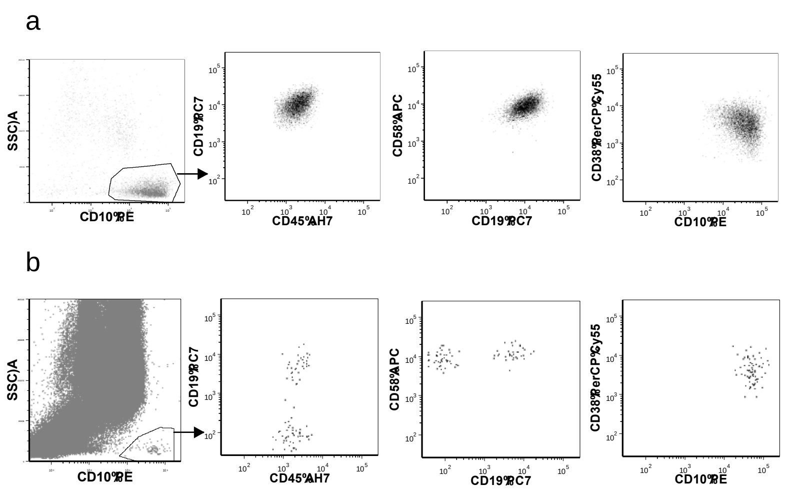

Supplementary Figure 1: Flow cytometry plots of bone marrow from patient ALL_H0082 pre-CAR (a) and post CAR (b). panels are gated on CD10+ SSC leukemic blasts.

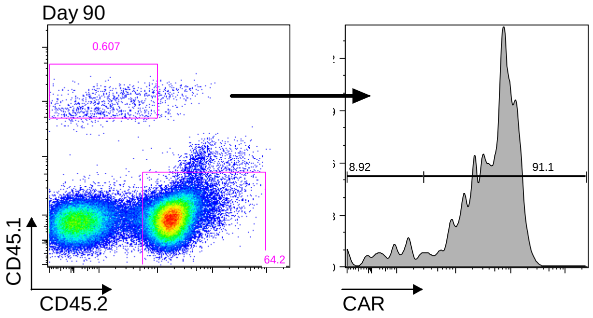

Supplementary Figure 2: C57Bl/6 mice injected with E2a:PBX leukemia and treated with mCD19 CAR were sacrifriced on dy +90. Splenocytes were harvested and stained for CD45 isoforms, T cell markers and protein L for CAR T cell detection, and analyzed by flow cytometry. The histogram on the right represents adoptively transferred T cells gated on CD45.1 isoform.

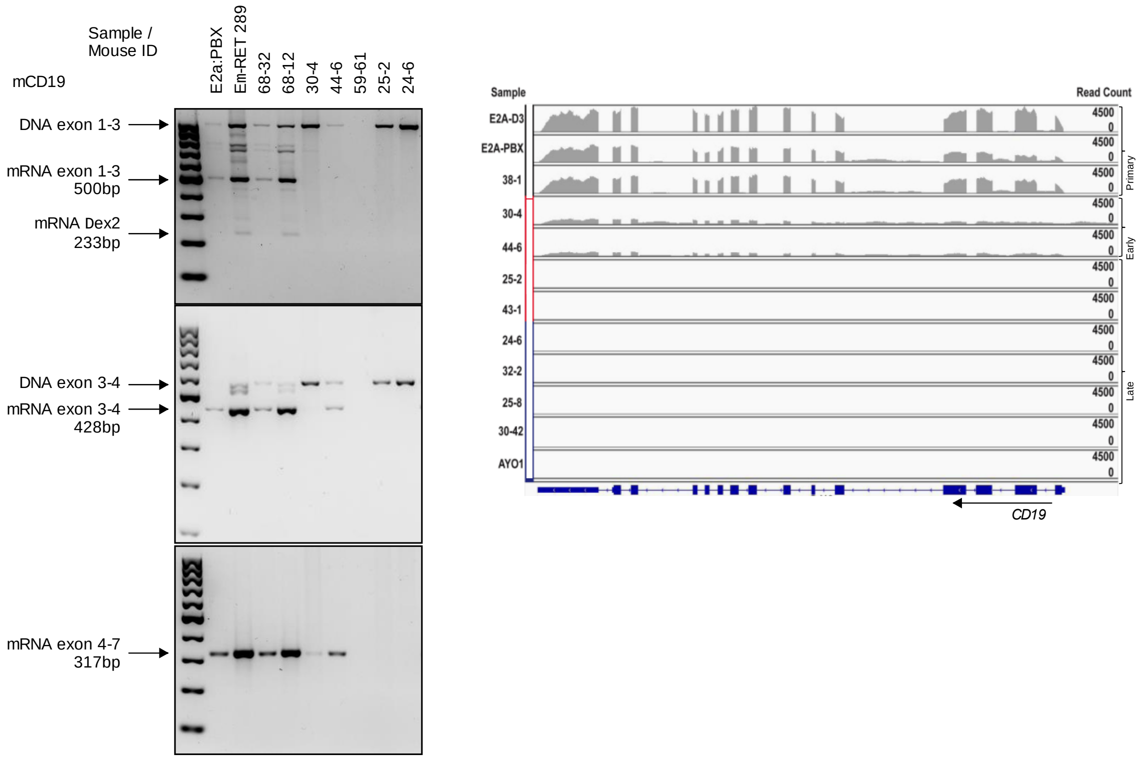

Supplementary Figure 3: RNA was extracted from parent E2a:PBX and Eu-RET cell lines, untreated splenocytes from E2a:PBX leukemic mice, and post-CD19-CAR CD19^{-} relapses. Left panel: CD19 exon specific primers were used in a PCR reaction and run on a gel. Locations of specific DNA and RNA sites are marked, and were confirmed by band sequencing. Right panel: screen-shot from RNA-sequencing directed at the CD19 transcript.

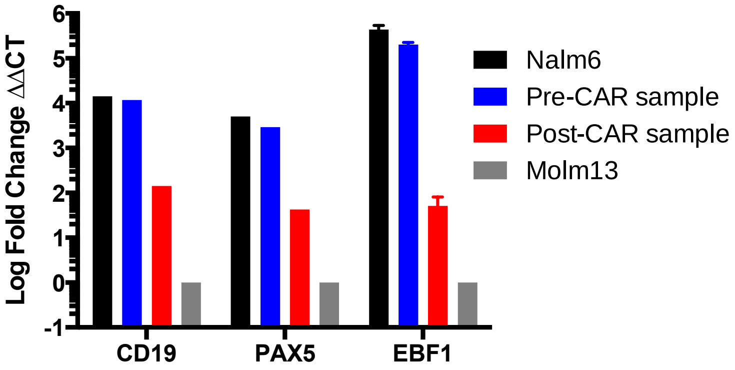

Supplementary Figure 4: mRNA expression of CD19, PAX5 and EBF1 by real-time RTPCR expression of NALM6 (pre-B ALL cell line, black bars), Molm13 (AML cell line, grey bars), and leukemic cells from patient ALL_H0140 (figure 1g-h) pre CD19-CAR (blue bars) and post CD19 CAR (red bars).

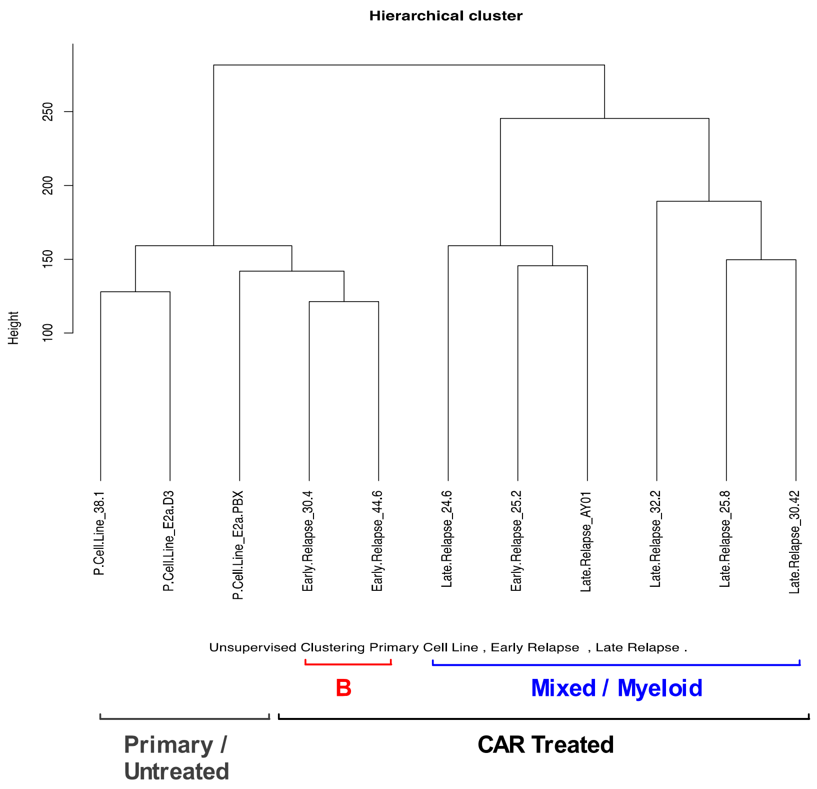

Supplementary Figure 5: Unsupervised hierarchical clustering on primary and post-CD19 CAR relapses of E2a:PBX murine leukemia, clustering 2 post-CAR sapmles with $CD19^{-}$ B-ALL (marked in red) with the primary/ untreated samples , and separate from the lineage switch samples (marked in blue).

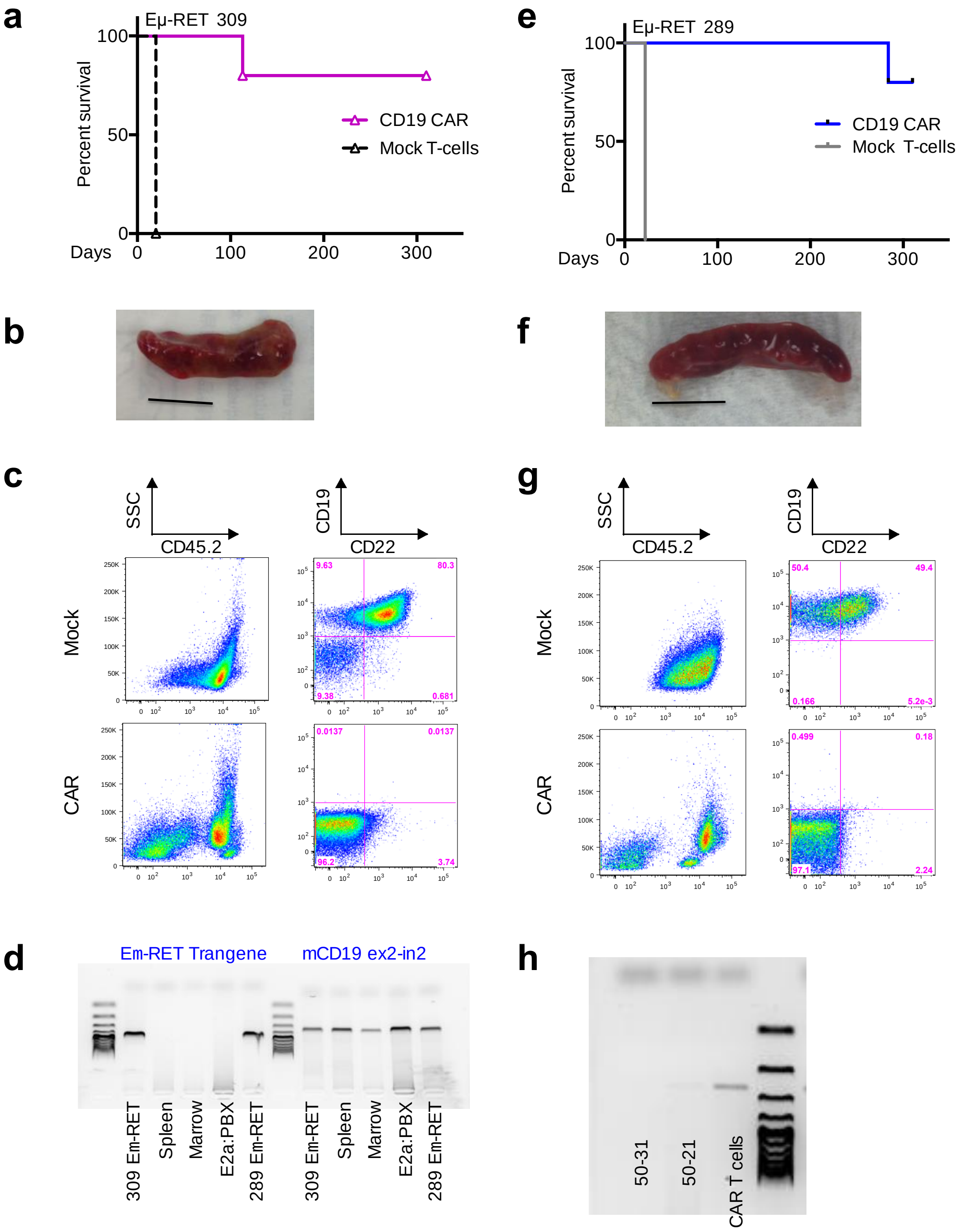

Supplementary Figure 6: Eμ-RET leukemia does not relapse following CD19 CAR and does not develop a lineage switch. (A-D) experiments performed on 309-Eμ-RET cell line. (a) survival curve of Balb/c mice treated as in figure 1a, with syngeneic 3x10^5 CD19 CAR T cells (n=5 mice per group). (b) Phenotype of a spleen of a mouse that died following CAR treatment, demonstrating sclerosis. Size bar is 1cm. (c) flow cytometry plots of mock treated (top) or CAR treated (bottom) Balb/C mice bearing 309-Eμ-RET leukemia. (d) PCR on genomic DNA for the Eμ-RET transgene was performed in tissues of the mouse found to die following CD19 CAR treatment. Controls include parent 309 and 289 Eμ-RET leukemia as positive controls, as well as E2a:PBX as a negative control. (e-g) similar graphs as in (a-c) on 289-Eu-RET ALL,

demonstrating late mortality in 1 of 5 mice following CD19 CAR, same phenotype of post-CAR mice with sclerotic spleen and absence of CD19+ cells or of a leukemic population. (h) PCR for the CD19 CAR was done in genomic DNA from mice dying late post CAR following 289 (50-21) or 309 (50-31) Eμ-RET leukemia. DNA extracted from CAR T cells used a control.

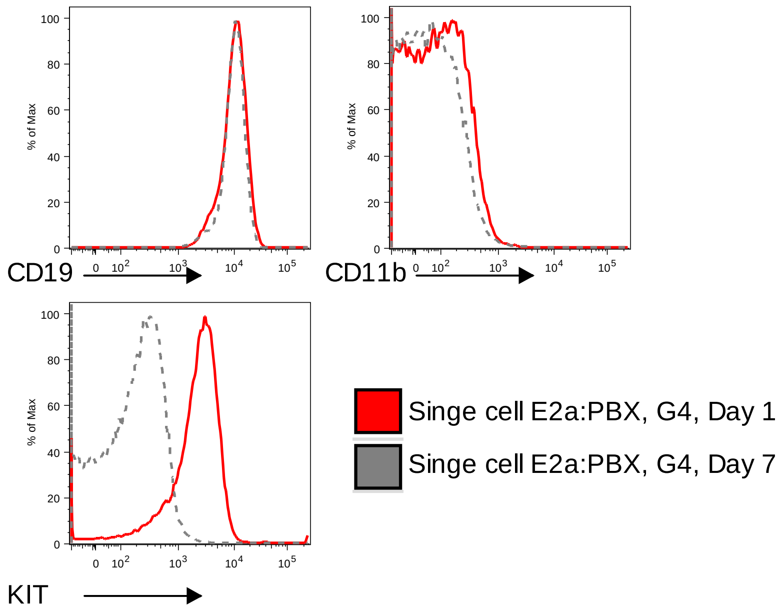

Supplementary Figure 7: Flow cytometry plots showing single cell cloning of E2a:PBX which resulted in a clone with high cKIT expression (red), with absence of cKIT positive cells following 1 week in culture (grey line).

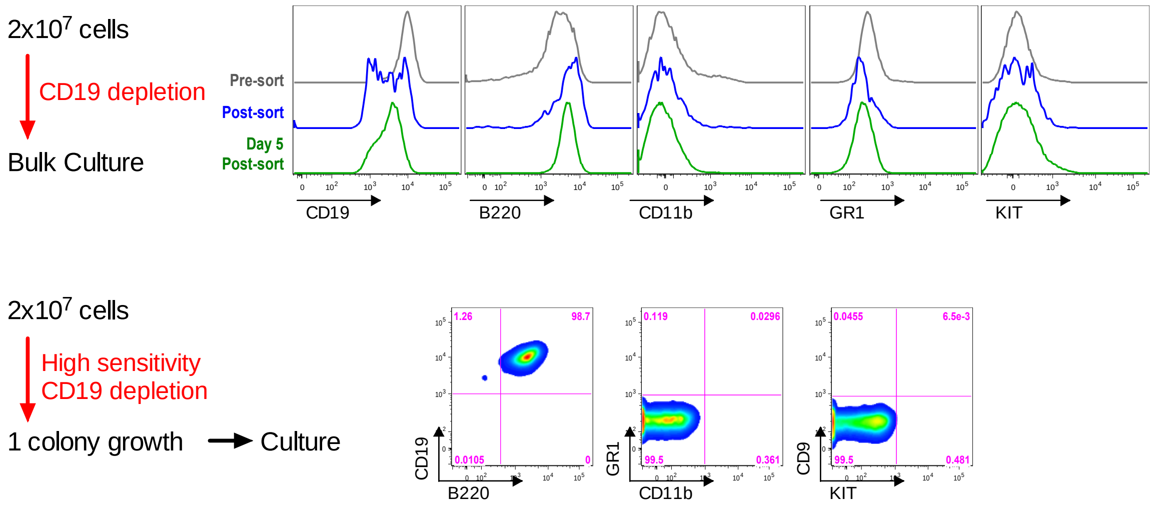

Supplementary Figure 8: Lack of myeloid cells upon antibody-based CD19 depletion of E2a:PBX cell lines. Upper panel: $20x10^7$ E2a:PBX cells were sorted using CD19 labeled magnetic beads in Miltenyi AutoMACS system, with the negative fraction kept in culture. Flow cytometry on pre-sorted cells (grey), immediate post-sort negative fraction (blue) and day 5 in culture following sort (green). Bottom panel: We conducted this experiment using a high sensitivity LD column for sorting, and single cell cloned immediately. Flow cytometry plots of the single colony that grew shown.

Murine CD19⁻ B-ALL post CD19 CAR relapse

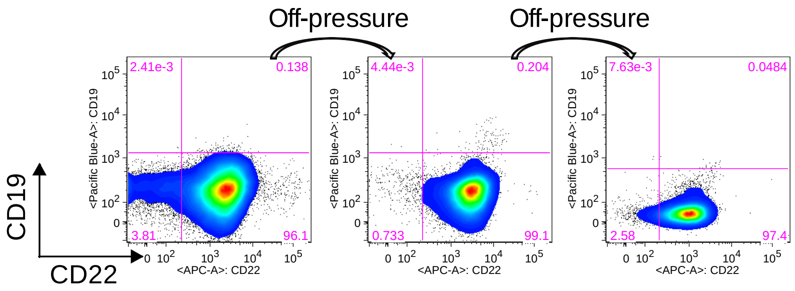

Human CD19⁻ B-ALL post CD19 CAR relapse

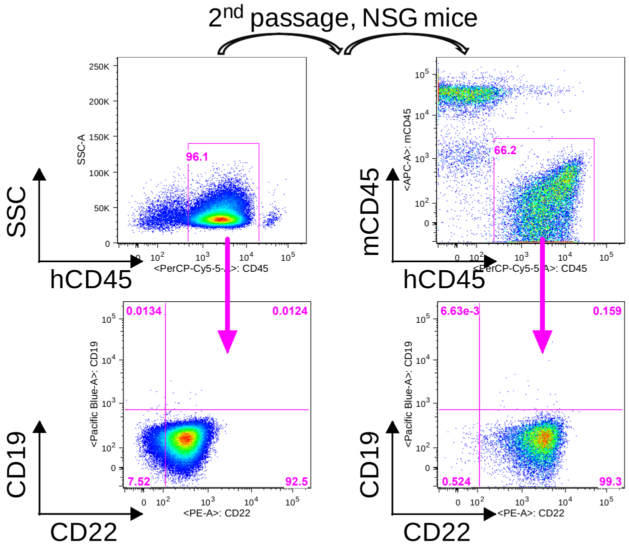

Supplementary Figure 9: Stability of CD19- B-ALL in murine and patient samples upon re-passaging off immune-pressure. Upper panel: Murine post-CAR sample 30-4 was passaged twice into mice off immunopressure, causing stable $CD19^-$ B-ALL in recipients. Lower panel: Patient sample with $CD19^-$ B –ALL following CD19 CAR was passaged twice in NSG mice. Flow cytometry analysis shown.

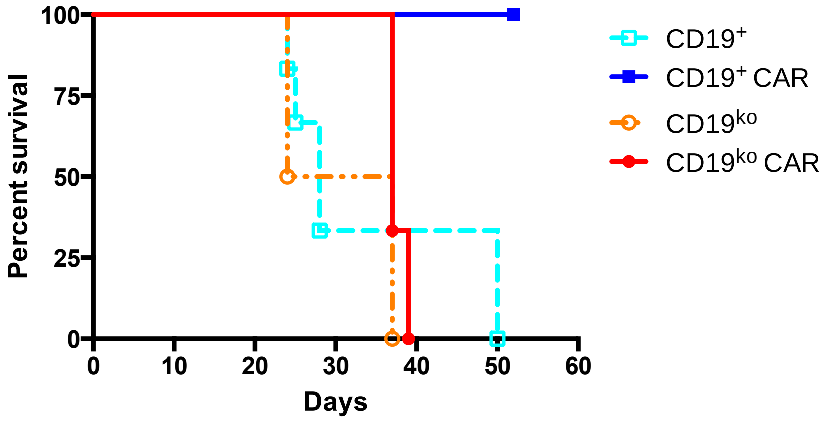

Supplementary Figure 10: Survival curve of E2a:PBX ALL injected mice untreated (cyan) or following CD19-CAR (blue); and of CD19^{ko}-E2a:PBX (generated using the CRISPR/CAS9 system) untreated (orange) or CD19-CAR treated (red).

Supplementary Table 1: Primary E2a:PBX cell lines and mouse relapse IDs

Sample	Description	Days to relapse	Immunophenotype	E2a:PBX1 PCR
E2a:PBX	Primary bulk cell line	NA	CD19^{+} CD22^{+} CD127^{+} B220^{+} BP1^{dim} CD93^{+} CD43^{+} KIT^{-} CD9^{-} Gr1^{-} CD11b^{-}	+
E2a:PBX B3	Single cell clone	NA	CD19^{+} CD22^{+} CD127^{+} B220^{+} BP1^{dim} CD93^{+} CD43^{+} KIT^{-} CD9^{-} Gr1^{-} CD11b^{-}	+
E2a:PBX D3	Single cell clone	NA	CD19^{+} CD22^{+} CD127^{+} B220^{dim} BP1^{+} CD93^{+} CD43^{+} KIT^{-} CD9^{-} Gr1^{-} CD11b^{-}	+
E2a:PBX G4	Single cell clone	NA	CD19^{+} CD22^{+} CD127^{+} B220^{+} BP1^{dim} CD93^{+} CD43^{+} KIT^{+} CD9^{-} Gr1^{-} CD11b^{-}	+
38-1	Mock treated *in vivo*	18	CD19^{+} CD22^{+} CD127^{+} B220^{+} BP1^{dim} CD93^{+} CD43^{+} KIT^{dim} CD9^{-} Gr1^{-} CD11b^{-}	+
68-32	Single cell clone *in vivo*, untreated	24	CD19^{+} CD22^{+} CD127^{+} B220^{+} BP1^{dim} CD93^{+} CD43^{+} KIT^{-} CD9^{-} Gr1^{-} CD11b^{-}	+
68-12	Single cell clone *in vivo*, untreated	25	CD19^{+} CD22^{+} CD127^{+} B220^{+} BP1^{dim} CD93^{+} CD43^{+} KIT^{-} CD9^{-} Gr1^{-} CD11b^{-}	+
78-51	Cy/ARA-C treated	20	CD19^{+} CD22^{+} CD127^{+} B220^{+} BP1^{+} CD93^{+} CD43^{+} KIT^{dim} CD9^{-} Gr1^{-} CD11b^{-}	+
78-53	Cy/ARA-C treated	28	CD19^{+} CD22^{+} CD127^{+} B220^{+} BP1^{dim} CD93^{+} CD43^{+} KIT^{-} CD9^{-} Gr1^{-} CD11b^{-}	+
78-54	Cy/ARA-C treated	31	CD19^{+} CD22^{+} CD127^{+} B220^{+} BP1^{+} CD93^{+} CD43^{+} KIT^{dim} CD9^{-} Gr1^{-} CD11b^{-}	+
78-55	Cy/ARA-C treated	41	CD19^{+} CD22^{+} CD127^{+} B220^{+} BP1^{+} CD93^{+} CD43^{+} KIT^{dim} CD9^{-} Gr1^{-} CD11b^{-}	+
30-4	CAR treated	46	CD19^{-} CD22^{+} CD127^{+} B220^{+} BP1^{-} CD93^{-} CD43^{+} KIT^{-} Gr1^{-} CD11b^{-}	+
44-6	CAR treated	47	CD19^{-} CD22^{+} CD127^{dim} B220^{+} BP1^{+} CD93^{+} CD43^{+} KIT^{-} CD9^{-} Gr1^{-} CD11b^{-}	+
59-61	CAR treated	58	CD19^{-} CD22^{+} CD127^{-} B220^{+} BP1^{-} CD93^{dim} CD43^{+} KIT^{-} CD9^{+} Gr1^{+} CD11b^{+}	+
25-2	CAR treated	76	CD19^{-} CD22^{+} CD127^{dim} B220^{dim} BP1^{+} CD93^{+} CD43^{+} KIT^{-} CD9^{dim} Gr1^{-} CD11b^{+}	+
43-1	CAR treated	76	CD19^{-} CD22^{+} CD127^{+} B220^{-} BP1^{dim} CD93^{+} CD43^{+} KIT^{dim} CD9^{+} Gr1^{+} CD11b^{+}	+
A001	CAR treated	90	CD19^{-} CD22^{-} CD127^{-} B220^{-} BP1^{dim} CD93^{dim} CD43^{+} KIT^{dim} CD9^{+} Gr1^{-} CD11b^{-}	+
24-6	CAR treated	110	CD19^{-} CD22^{-} CD127^{-} B220^{-} BP1^{-} CD93^{+} CD43^{+} KIT^{dim} CD9^{dim} Gr1^{dim} CD11b^{+}	+
30-42	CAR treated	121	CD19^{-} CD22^{-} CD127^{-} B220^{-} BP1^{-} CD93^{-} CD43^{+} KIT^{-} CD9^{+} Gr1^{-} CD11b^{-}	+
32-2	CAR treated	160	CD19^{-} CD22^{-} CD127^{-} B220^{-} BP1^{-} CD93^{dim} CD43^{+} KIT^{dim} CD9^{+} Gr1^{+} CD11b^{+}	+
25-8	CAR treated	210	CD19^{-} CD22^{-} CD127^{dim} B220^{-} BP1^{-} CD93^{+} CD43^{+} KIT^{+} Gr1^{-} CD11b^{-}	-
48-21	CAR treated	225	CD19^{-} CD22^{-} CD127^{-} B220^{-} BP1^{-} CD93^{dim} CD43^{+} KIT^{-} CD9^{-} Gr1^{-} CD11b^{-}	-
48-22	CAR treated	232	CD19^{-} CD22^{-} CD127^{-} B220^{-} BP1^{-} CD93^{dim} CD43^{+} KIT^{+} CD9^{-} Gr1^{+} CD11b^{-}	-
21-1	CAR treated	268	CD19^{-} CD22^{-} CD127^{-} B220^{-} BP1^{-} CD93^{-} CD43^{+} KIT^{-} Gr1^{+} CD11b^{+}	+
47-24	21-1, *in vivo* passage	NA	CD19^{-} CD22^{-} CD127^{-} B220^{-} BP1^{-} CD93^{-} CD43^{+} KIT^{-} Gr1^{+} CD11b^{+}	+
53-41	44-6, *in vivo* passage	NA	CD19^{-} CD22^{+} CD127^{dim} B220^{+} BP1^{+} CD93^{+} CD43^{+} KIT^{-} CD9^{-} Gr1^{-} CD11b^{-}	+
53-42	44-6, *in vivo* passage	NA	CD19^{-} CD22^{+} CD127^{dim} B220^{+} BP1^{+} CD93^{+} CD43^{+} KIT^{-} CD9^{-} Gr1^{-} CD11b^{-}	+

Supplementary Table 2: Primers

Name	Forward (5'→3')	Reverse (5'→3')
E2a:PBX transgene	GCACAACCACGCGGCCC	CCACGCCTTCCGCTAACAGC
Eu-RET transgene	CATTGGGGACACATTGAGCAG	TAGCAGTGGATGCAGAAGGCAGAC
mCD19 ex2-in2	GGAGAGCACCCGGTCAGAGAG	CCTTGTCTGTCTGTCTGTCTGTCTGTC
mCD19 ex1-3	CCCAGCATCCTCTGCGCAG	GAAGTGGACCTGTGGCTCCC
mCD19 ex3-4	CAGGGACCTGGACTGTGACC	CCTTCACGTGCCTCTCGATGG
mCD19 ex4-7	GGGTCTCTTCTGCTTCTGCCC	CCGTACTGGTTCTGGGTCCCG
mCD19 ex4-6	GGCACCTATTATTGTCTCCG	GGGTCAGTCATTCGCTTC
mPAX5	GCATAGTGTCTACAGGCTCCG	GATGGGTTCCGTGGTGGT
mEBF1	CGAGATCATGTGCAGCCGC	CCTGGAATCTCCGCATGTCACG
hCD19 ex1-3	CACCCCCATGGAAGTCAGGC	GGTGAGGTCCTGGCTGAGG
hCD19 ex3-4	GGAGGGAGAGCCTCCGTG	GGGCAACAACAGACCCGTCTC
hCD19	GCACTGGCTGCTGAGGACT	CTTTTCCTCCTCAGGACCAGGG
hPAX5	GGGAGATCAGGGACCGGC	GCTGTGACTGGAAGCTGGGAC
hEBF1	TGCCGAGTCTTGCTCACAC	CATTGACTGTCGTAGACACCAC
mCD19_CRISPR	CACCGTGACGTCTGAAGCATTCCAC	AAACGTGGAATGCTTCAGACGTCAC
mEBF1_CRISPR	CACCGGATTGAGAGGACGGCCTTTG	AAACCAAAGGCCGTCCTCTCAATCC
mPAX5_CRISPR	CACCGGGGCAAGTTCCACTATCCTT	AAACAAGGATAGTGGAACTTGCCCC
