# Supplementary material for: CD19 CAR immune pressure induces B-precursor acute lymphoblastic leukaemia lineage switch exposing inherent leukaemic plasticity
Source: Nat Commun. 2016 Jul 27;7:12320. doi: 10.1038/ncomms12320 (PMC4974466; doi:10.1038/ncomms12320)
Supplement: Peer Review File [file ncomms12320-s2.pdf]

Editorial Note: this manuscript has been previously reviewed at another journal that is not operating a transparent peer review scheme. This document only contains reviewer comments and rebuttal letters for versions considered at Nature Communications.
